# Chloroplast proteome analysis of *Nicotiana tabacum* overexpressing TERF1 under drought stress condition

**DOI:** 10.1186/s40529-018-0239-5

**Published:** 2018-10-29

**Authors:** Wei Wu, Yanchun Yan

**Affiliations:** Graduate School of Chinese Academy of Agricultural Sciences, No. 12 Zhongguancun South St., Haidian District, Beijing, 100081 People’s Republic of China

**Keywords:** Chloroplast proteome, Drought stress, TERF1, Ethylene signaling pathway

## Abstract

**Background:**

Chloroplast is indispensable for plant response to environmental stresses, growth and development, whose function is regulated by different plant hormones. The chloroplast proteome is encoded by chloroplast genome and nuclear genome, which play essential roles in plant photosynthesis, metabolism and other biological processes. Ethylene response factors (ERFs) are key transcription factors in activating the ethylene signaling pathway and plant response to abiotic stress. But we know little about how ethylene regulates plastid function under drought stress condition. In this study we utilized tobacco overexpressing tomato ethylene responsive factor 1 (TERF1), an ERF transcription factor isolated from tomato, to investigate its effects on the plastid proteome under drought stress condition by method of iTRAQ technology.

**Results:**

Results show that TERF1 represses the genes encoding the photosynthetic apparatus at both transcriptional and translational level, but the genes involved in carbon fixation are significantly induced by TERF1. TERF1 regulates multiple retrograde signaling pathways, providing a new mechanism for regulating nuclear gene expression. TERF1 also regulates plant utilization of phosphorus (Pi) and nitrogen (N). We find that several metabolic and signaling pathways related with Pi are significantly repressed and gene expression analysis shows that TERF1 significantly represses the Pi transport from root to shoot. However, the N metabolism is upregulated by TERF1 as shown by the activation of different amino acids biosynthesis pathways due to the induction of glutamine synthetase and stabilization of nitrate reductase although the root-to-shoot N transport is also reduced. TERF1 also regulates other core metabolic pathways and secondary metabolic pathways that are important for plant growth, development and response to environmental stresses. Gene set linkage analysis was applied for the upregulated proteins by TERF1, showing some new potential for regulating plant response to drought stress by TERF1.

**Conclusions:**

Our research reveals effects of ethylene signaling on plastid proteome related with two key biological processes, including photosynthesis and nutrition utilization. We also provide a new mechanism to regulate nuclear gene expression by ERF1 transcription factor through retrograde signals in chloroplast. These results can enrich our knowledge about ERF1 transcription factor and function of ethylene signaling pathway.

**Electronic supplementary material:**

The online version of this article (10.1186/s40529-018-0239-5) contains supplementary material, which is available to authorized users.

## Background

Ethylene is a gaseous phytohormone that plays vital roles in plant growth, development as well as response to abiotic and biotic stresses. ERF1 protein is a key transcription factor for activating the ethylene signaling pathway by binding the GCC box and other *cis*-elements in the promoters of ethylene-responsive genes, which is an ideal candidate gene for improving plant tolerance of different abiotic stresses (Kavas et al. 2015).

Chloroplast is an organelle derived from the cyanobacteria and its proteome is encoded by nuclear genome and its own genome. Apart from the key function of photosynthesis, chloroplast is also responsible for the biosynthesis of different metabolites, including amino acids, vitamins, fatty acids, nucleotides, phytohormones as well as assimilation of sulfur and nitrogen (Daniell et al. 2016). Chloroplast has been found to regulate plants’ response to different environmental stresses through organellar signaling pathways because it can act as environment sensor (Kmiecik et al. 2016).

At transcriptional level different phytohormones have been found to regulate chloroplast transcription. Abscisic acid (ABA) represses the plastid encoded RNA polymerase (PEP) and nuclear encoded RNA polymerase (NEP), leading to the reduced transcript level of most chloroplast genes, which can be minimized by cytokinin (Yamburenko et al. 2015). Methyl jasmonate (MeJA), gibberellic acid [GA(3)], an auxin (indole-3-acetic acid, IAA) repress both transcription and transcript accumulation of chloroplast genes, while a brassinosteroid (24-epibrassinolide, BR) can counteract these effects (Zubo et al. 2011).

Additionally, characterization of chloroplast proteome changes under environmental stress condition attracted the attention of the researchers. 31 chloroplast proteins were found to be differentially regulated by drought stress, which are mainly enriched in the Gene Ontology (GO) category of “energy” (Zubo et al. 2011). Kosmala et al. identified 10 differentially accumulated chloroplast proteins in high-drought-tolerant and low-drought-tolerant of *Festuca arundinacea* (Kosmala et al. 2012). Uberegui et al. revealed the function of Executer proteins in plant chloroplast response to increased light condition (Uberegui et al. 2015). Wang et al. studied the chloroplast proteome changes in mangrove under different salt stress conditions (Wang et al. 2013). Although these studies have revealed some mechanisms in chloroplast response to environmental stresses, most of these proteomics studies utilized 2D-Gel method, which restricted coverage of the chloroplast proteome and the accuracy of protein quantification. Additionally, there is little report on how ethylene regulate chloroplast proteome under environmental stress conditions.

TERF1, an ERF1 protein isolated from tomato, can activate ethylene signaling pathway through binding the GCC box and dehydration responsive element (DRE) in the promoters of the target genes (Huang et al. 2004). Overexpression of TERF1 can significantly improve tobacco tolerance of abiotic stress (Huang et al. 2004), however the detailed mechanism of TERF1 in improving the plant tolerance of drought stress is not clear. In this study we compared the chloroplast proteome between WT tobacco and tobacco overexpressing TERF1 under natural dehydration condition by the isobaric tags for relative and absolute quantification (iTRAQ) method, aiming to further reveal the function of TERF1 and ethylene in plant response to drought stress.

## Results

### TERF1 significantly changes the chloroplast proteome under drought stress condition

After drought stress was exerted we measured the relative water content (RWC) of the WT and transgenic tobacco and results showed that the RWC of WT tobacco decreased faster than the transgenic ones after 10 days of dehydration and at 20 days of dehydration the RWC of WT tobacco decreased by more than 35% and the TERF1 tobacco decreased only about 18% (Additional file 1: Figure S1), indicating that TERF1 significantly reduced water loss under drought stress condition. We compared the chloroplast proteome between TERF1 and WT tobacco after 20 days of dehydration when the RWC of WT tobacco was significantly lower than TERF1 tobacco.

We identified 13,049 unique peptides (Additional file 2: Table S9), which were searched against the UniProt database of *Nicotiana tabacum* and 4732 proteins were identified and quantified (at least two unique peptide with high confidence), among which 4694 proteins are encoded by nuclear genome (Additional file 3: Table S2) and 38 proteins are encoded by chloroplast genome (Additional file 9: Table S7). A significant difference is observed in chloroplast proteome between WT and TERF1 tobacco supported by principal component analysis (PCA) (Additional file 4: Figure S2).

We use more than 1.20-fold (P < 0.05) or less than 0.83-fold (P < 0.05) cutoff to identify the differentially expressed proteins (DEPs), of which 189 proteins are significantly induced (Additional file 5: Table S3) and 273 proteins are significantly repressed (Additional file 6: Table S4). In order to dissect the function of the DEPs the KEGG pathway enrichment analysis was applied and the significantly enriched pathways are shown in Tables 1 and 2. The results of gene ontology (GO) enrichment analysis for the DEPs are listed in Additional file 7: Table S5 and Additional file 8: Table S6, respectively.

**Table 1:** KEGG pathway enrichment for the upregulated DCPs

Term	ID	Number of the enriched proteins (%)	P-value
Metabolic pathways	ko01100	61 (32%)	0.009696835
Biosynthesis of secondary metabolites	ko01110	41 (21%)	0.005100797
Biosynthesis of amino acids	ko01230	19 (10%)	0.002111965
Terpenoid backbone biosynthesis	ko00900	5 (3%)	0.012298651
Fatty acid biosynthesis	ko00061	5 (3%)	0.013993754
mRNA surveillance pathway	ko03015	5 (3%)	0.05547601
Pantothenate and CoA biosynthesis	ko00770	4 (2%)	0.007031075
Valine, leucine and isoleucine biosynthesis	ko00290	4 (2%)	0.010395488
One carbon pool by folate	ko00670	4 (2%)	0.014658739
Cyanoamino acid metabolism	ko00460	4 (2%)	0.029672314
Isoquinoline alkaloid biosynthesis	ko00950	3 (2%)	0.021900026
Biotin metabolism	ko00780	3 (2%)	0.026505785

**Table 2:** KEGG pathway enrichment for the downregulated DCPs

TERM	ID	Number of the enriched proteins (%)	P-value
Metabolic pathways	ko01100	62 (23%)	0.007587671
Photosynthesis	ko00195	15 (5%)	1.66E−07
Photosynthesis-antenna proteins	ko00196	8 (3%)	1.22E−07
Glycerophospholipid metabolism	ko00564	6 (2%)	0.000985438
Phenylpropanoid biosynthesis	ko00940	6 (2%)	0.007947381
Phosphatidylinositol signaling system	ko04070	4 (1%)	0.004652457
Ether lipid metabolism	ko00565	3 (1%)	0.002725805
ABC transporters	ko02010	2 (1%)	0.005861127
Diterpenoid biosynthesis	ko00904	2 (1%)	0.002013018

### TERF1 exerts different effects on the expression of the genes in photosynthetic light and dark reaction

Chloroplast proteome are encoded by nuclear genome and chloroplast genome together, among which 98 proteins are encoded by tobacco chloroplast genome. The genes encoded by chloroplast genome are related with photosynthetic apparatus, the chloroplast genetic system, and other functions (Shimada and Sugiura 1991). 38 chloroplast genome encoded proteins were identified and quantified in our proteomics analysis, among which 27 proteins were repressed by TERF1 and most of these proteins are components of photosynthesis apparatus (Additional file 9: Table S7).

We also assessed the transcript level of the 79 genes encoded by chloroplast genome with confirmed function, showing that 61 genes were repressed and only 18 were induced by TERF1 (Additional file 10: Table S8). The significantly repressed genes encode subunits of PSI, PSII, NADH dehydrogenase, ATP synthase, cytochrome b6/f and ribosome proteins (Additional file 10: Table S8). As the bacteria-type 70S ribosome is responsible for protein biosynthesis in chloroplast (Tiller and Bock 2014), the significant repression of the *rps* and *rpl* genes by TERF1 contribute to the repression of protein biosynthesis in chloroplast.

Additionally TERF1 negatively regulates RNA editing in chloroplast as shown by the significant repression of Multiple Organellar RNA Editing Factor 8 (MORF8) (No. 122, Additional file 6: Table S4). MORF8 participates in chloroplast RNA editing as well as 8 mitochondrial genes editing (Glass et al. 2015; Huang et al. 2017). Repression of MORF8 will negatively affect chloroplast and plant growth and development (Glass et al. 2015).

TERF1 also represses many genes encoded by nuclear genome participating in the process of light harvesting, light reaction and photosynthetic electron transport (Additional file 8: Table S6). Additionally, we also found FtsH5, a thylakoid membrane-bound metalloprotease, was significantly repressed (No. 32, Additional file 6: Table S4), which will impair the D1 protein degradation and finally leads to photoinhibition (Kato et al. 2009). Finally, two genes involved in the chlorophyll biosynthesis, Glutamate–tRNA ligase (GluRSAt) and Protoporphyrinogen oxidase (PPO2) (No. 91 and No. 200, Additional file 6: Table S4), were also significantly repressed by TERF1. Repression of GluRSAt and PPO2 will reduce the accumulation of Glutamyl-tRNA^Glu^, starting substrate of tetrapyrroles biosynthesis, and Protoporphyrin IX, the last common precursor for chlorophyll and heme biosynthesis, respectively.

Although many genes involved in photosynthetic light reactions were repressed by TERF1, seven GO terms related with carbon fixation are significantly enriched for the upregulated DEPs, including “GO 0034637: cellular carbohydrate biosynthetic process”, “GO 0033692: cellular polysaccharide biosynthetic process”, “GO 0042732: d-xylose metabolic process”, “GO 0009250: glucan biosynthetic process”, “GO 0000271: polysaccharide biosynthetic process”, “GO 0019252: starch biosynthetic process”, GO 0005982: starch metabolic process” (Additional file 7: Table S5), indicating that carbon fixation in TERF1 tobacco is significantly upregulated.

According to the protein expression profile, several key genes involved in starch biosynthesis are significantly induced by TERF1. Protein Targeting To Starch (PTTS) is upregulated by more than 32% by TERF1, which localise granule-bound starch synthase to starch granules and is responsible for normal amylose synthesis (No. 125, Additional file 5: Table S3) (Seung et al. 2015). ADP-Glucose Pyrophosphorylase (ApL3), catalyzing the first and limiting step in starch biosynthesis (Sulmon et al. 2011), is significantly induced by 26% in TERF1 tobacco (No. 66, Additional file 5: Table S3). Starch Synthase 2 (SS2), involved in amylopectin biosynthesis (Zhang et al. 2008), is induced by more than 25% in TERF1 tobacco. The upregulation of the carbon fixation in TERF1 tobacco will also contribute to the repression of the genes involved in photosynthetic light reaction (Hausler et al. 2014).

### TERF1 regulates four key chloroplast metabolic pathways

According to the KEGG pathway enrichment analysis, the pathway of “valine, leucine and isoleucine biosynthesis”, “biosynthesis of amino acids” and “cyanoamino acid metabolism” are significantly enriched for the upregulated DEPs in TERF1 tobacco (Table 1). Another five GO terms related with amino acids metabolism (GO 1901607: alpha-amino acid biosynthetic process, GO 0006541: glutamine metabolic process, GO 0006545: glycine biosynthetic process, GO 0009070: serine family amino acid biosynthetic process, GO 0006556: S-adenosylmethionine biosynthetic process) are also significantly enriched for the upregulated DEPs (Additional file 7: Table S5), indicating that N assimilation is improved by TERF1 (Li et al. 2017).

**Fig. 1:**
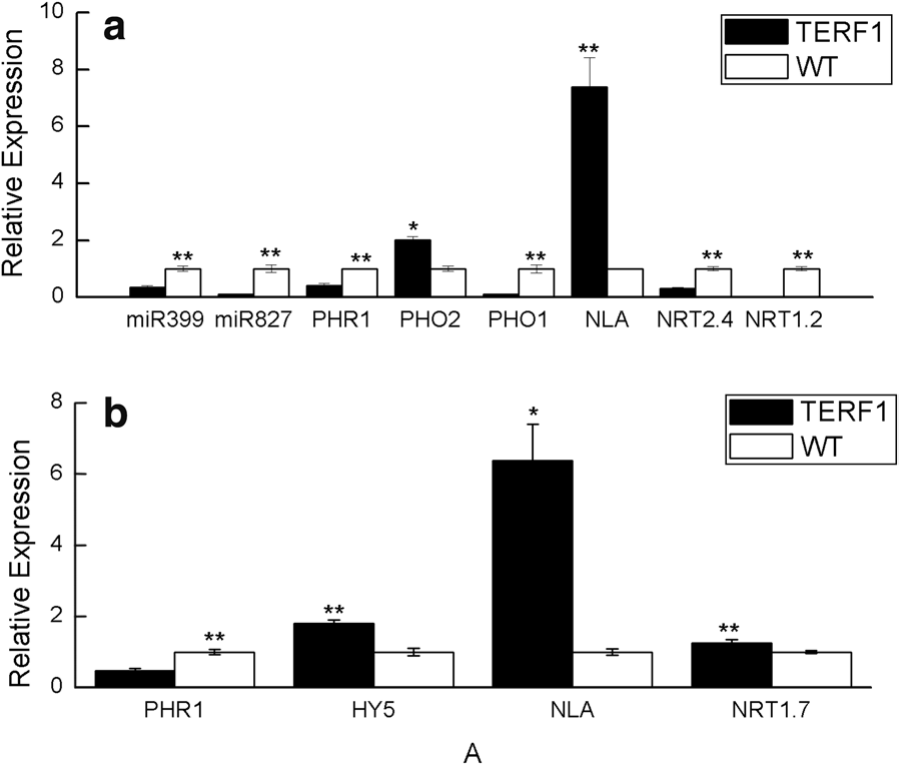
Quantitative real-time polymerase chain reaction (qRT-PCR) for expression analysis of the genes related N and Pi metabolism in root (**a**) and leaf (**b**). Means ± SDs, n = 3; * and **significantly different at 5% and 1% level of probability, respectively

Gene expression results show that TERF1 significantly represses *NRT2.4* and *NRT1.2* (Fig. 1a). NRT2.4 and NRT1.2 encode high-affinity and low-affinity nitrate transporter, respectively (Filleur et al. 2001), indicating that TERF1 restricts the nitrate uptake in the root under drought stress condition. Moreover, the significant induction of NLA in leaf will degrade NRT1.7 although *NRT1.7* transcript level is slightly induced (Fig. 1b). NRT1.7 is responsible for source-to-sink nitrate remobilization and negatively regulates plant adaptation to limited nitrogen (Liu et al. 2017a, b), indicating that TERF1 also limits the N remobilization under drought stress condition.

However, at protein level we find that TERF1 significantly upregulates the glutamine synthetase (GS) (No. 67, Additional file 5: Table S3), a key enzyme for improving N use efficiency (NUE) through converting ammonium into glutamine (Wang et al. 2018). And two 14-3-3 proteins are significantly repressed by TERF1 (No. 2 and No. 3, Additional file 6: Table S4), which can stabilize nitrate reductase (NR), a key enzyme for reducing nitrate in chloroplast (Miller et al. 2008). N assimilation in chloroplast also correlates with the TCA cycle in the mitochondria because it can provide C skeleton for de novo ammonium assimilation. We find that the dicarboxylate transporter (No. 54, Additional file 5: Table S3) is significantly induced by TERF1, which can promote the transport of 2-oxoglutarate into the chloroplast, an important C skeleton for ammonium assimilation by GS (Taniguchi and Taniguchi et al. 2002). 2-oxoglutarate also regulates GS activity at transcriptional or post-translational level (Galvez et al. 1999). Upregulation of GS and stabilization of NR provide a possible mechanism for the enhanced N assimilation efficiency by TERF1. As NUE is composed of N uptake efficiecy and N utilization efficiency (Wang et al. 2018), we propose that TERF1 significantly improve N utilization efficiency.

As described above many genes related with photosynthesis are significantly repressed we speculate that TERF1 may repress Phosphorus (Pi) utilization because Pi is an essential nutrient for plant photosynthesis (Zhang et al. 2014). Additionally we also find several pathways related with Pi metabolism or signaling pathway are significantly enriched for the downregulated proteins, including phosphatidylinositol signaling system, glycerophospholipid metabolism, inositol phosphate metabolism (Table 2) and phosphatidylcholine metabolic process (GO: 0046470) (Additional file 8: Table S6). Additionally, repression of the genes encoding subunits of ATP synthase described above may also result from the repression of Pi deficiency (Zhang et al. 2014).

Based on the above results we compared the expression of genes responsible for root-to-shoot Pi transport between WT and TERF1 tobacco. Phosphate Starvation Response 1 (PHR1), a conserved MYB transcription factor for regulating plant Pi starvation response, is significantly repressed by TERF1, which can cause the repression of miR399 and miR827 (Fig. 1a) (Chien et al. 2017). Repression of miR399 can upregulate the expression of *Phosphate 2* (*PHO2*) (Fig. 1a), a ubiquitin-conjugating E2 enzyme for degrading Pi transporters phosphate 1 (PHO1) and phosphate transporter 1 (PHT1) (Chien et al. 2017). Moreover, the transcript level of *PHO1* is also significantly reduced by TERF1 (Fig. 1a). Repression of miR827 can lead to the upregulation of *NLA* (Fig. 1a), another ubiquitin E3 ligase for degrading the PHT1 (Chien et al. 2017). So TERF1 represses the expression of the Pi transporters at both transcriptional and posttranslational level, indicating that it can significantly reduce the root-to-shoot Pi uptake. In leaf *PHR1* is also significantly repressed by TERF1, which may result from the upregulation of HY5 as it can repress the expression of PHR1 activated by light and ethylene signaling pathway (Fig. 1b) (Liu et al. 2017a, b).

Sulfur assimilation is upregulated by TERF1 as shown by the two GO term, “sulfur compound biosynthetic process” and “sulfur compound metabolic process”, are significantly enriched for the upregulated proteins in TERF1 tobacco. The enriched proteins for the sulfur metabolism correlates with the biosynthesis of S-adenosylmethionine (SAM) (No. 2 and No. 143, Additional file 5: Table S3), thiamine (vitamin B1) (No. 158, Additional file 5: Table S3), biotin (vitamin H) (No. 35, Additional file 5: Table S3). Accumulation of SAM provides substrates for the biosynthesis of plant hormones, polyamines, and defense metabolites (Kusano et al. 2010). Acetyl CoA carboxylase is significantly upregulated by TERF1 (No. 22 and No. 23, Additional file 5: Table S3) and biotin is an important co-factor for it (Nikolau et al. 2003), which contribute to the significant upregulation of fatty acid biosynthesis as shown in Table 1. Biosynthesis of thiamine contributes to the carbon fixation shown in Additional file 7: Table S5, glycolysis and citric acid cycle (Martinis et al. 2016).

Finally we find that TERF1 can increase the supply of one-carbon. Serine accumulation in TERF1 tobacco provides the substrate for one-carbon metabolism. And three serine hydroxymethyltransferase are significantly upregulated by TERF1 (No. 147–149, Additional file 5: Table S3), which convert serine into glycine and 5,10-methylenetetrahydrofolate that are important for plant growth and development (Zhang et al. 2010). And the upregulation of SAM also provide an important source of one-carbon.

### TERF1 regulates the retrograde signals in chloroplast

Retrograde signaling is defined by chan et al. as a process in which a stimulus perturbs chloroplast homeostasis and gives rise to one or more retrograde signals that alter transcriptional through to posttranslational processes and ultimately regulate chloroplast function (Chan et al. 2016).

Transcription as well as protein biosynthesis in chloroplast can act as retrograde signals to regulate the nuclear genes expression, especially the photosynthesis-associated nuclear genes (PhANGs) (Chan et al. 2016). The repression of transcription and protein synthesis in chloroplast by TERF1 described above are important signals for the downregulation of proteins enriched with pathway of “photosynthesis” and “photosynthesis-antenna proteins” described in Table 2 and Additional file 6: Table S4 because chloroplast gene expression need to coordinate with their counterparts encoded by nuclear genome performing the same function (Nott et al. 2006).

Methylerythritol cyclodiphosphate (MEcPP), an isoprenoid precursor in chloroplast, is the second retrograde signal regulated by TERF1. TERF1 significantly induces 1-hydroxy-2-methyl-2-(E)-butenyl-4-diphosphate synthase (HDS) (No. 16, Additional file 5: Table S3), which can convert MEcPP into hydroxymethylbutenyl diphosphate (HMBPP) (Chan et al. 2016). The decrease of MEcPP can repress the expression of hydroxyperoxide hyase (HPL) protein (No. 109, Additional file 6: Table S4) and salicylic acid (SA) accumulation (Xiao et al. 2012). HPL protein also correlates with the chloroplast retrograde signal of oxylipins, derived from the oxidized linoleic acid and linolenic acid in chloroplast. The repression of HPL protein will cause the accumulation of 12-oxophytodienoic acid (12-OPDA) as well as the biosynthetic precursor for jasmonates (de Souza et al. 2017). Additionally 12-OPDA itself can also act as signal to regulate nuclear gene expression through interaction with the transcription factors binding the TGACG motif, which regulates the stomatal closure in response to drought (de Souza et al. 2017).

Thioredoxins (Trxs) are a large protein family acting as redox regulators to regulate the structure and function of plant proteins through catalyzing the reduction of disulfide bonds in them. (Holmgren 1985). As Trxs can lead to changes in sugars, intermediates of chlorophyll synthesis, ROS, chloroplast protein synthesis, they have been proposed to be involved in the retrograde signaling to nucleus (Brunkard et al. 2015). Two chloroplast-localized Trxs (Trx f1 and HCF164) are significantly repressed by TERF1 (No. 230 and No. 233, Additional file 6: Table S4), while CDSP32 is significantly induced (No. 159, Additional file 5: Table S3). Trx f1 regulates multiple enzymes involved in Calvin–Benson cycle and starch synthesis (Thormahlen et al. 2013). Repression of Trx f1 in TERF1 will attenuate the starch biosynthesis (Thormahlen et al. 2013), indicating that sucrose signaling may be activated by TERF1. HCF164 is involved in the biosynthesis and assembly of cyt b6/f complex (Gabilly et al. 2010), providing an important mechanism for the downregulation of the genes encoding cyt b6/f complex by TERF1 (Additional file 6: Table S4 and Additional file 10: Table S8), indicating that it is involved in the plastid gene expression retrograde signaling. CDSP32 protects photosynthetic apparatus against oxidative damage, showing that it may participate in the retrograde signaling of oxidative stress (Broin et al. 2002).

Tetrapyrroles are the fourth kind of retrograde signals regulated by TERF1. As described above the repression of GluRSAt and PPO2 can inhibit the biosynthesis of tetrapyrroles, which can also lead to the repression of PhANGs (Nott et al. 2006).

The last retrograde signal regulated by TERF1 results from the repression of STN7, which phosphorylates LHCII to mediate state transitions and acts as sensor of the plastoquinol (PQH2)/plastoquinone (PQ) redox state (Chan et al. 2016). PQ/PQH2 redox state can regulate at least 750 nuclear genes expression through STN7 (Chan et al. 2016), so the repression of STN7 partially blocks the redox cues from chloroplast.

### Gene set linkage analysis of the DEPs

**Fig. 2:**
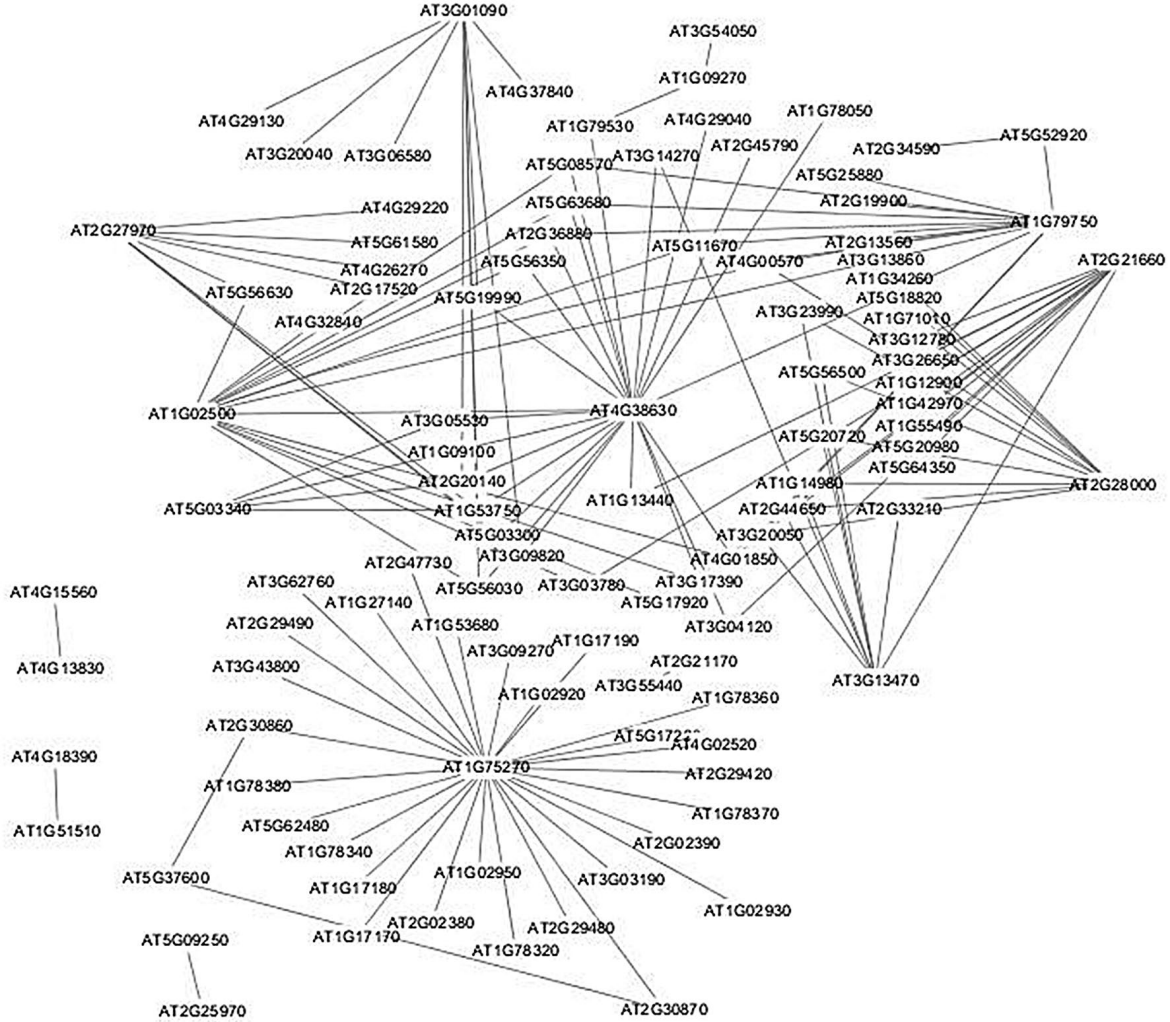
Protein–protein interaction network of the upregulated DEPs

Gene set linkage analysis (GSLA) interpretates the differentially expressed genes functional impact on biological processes in Arabidopsis with the aim to solve the problems of “no annotation” or “only conceptually general terms (such as “GO 0007165: signal transduction”)” in GO enrichment analysis (Yao et al. 2018). We first searched the corresponding orthologous genes of the DEPs in Arabidopsis and conducted GSLA analysis. As no significant GO term is enriched for the downregulated proteins, we only analyzed the PPI network of upregulated proteins based on GO enrichment and topology characteristics of the network (Fig. 2).

According to PPI network described in Fig. 2, most of the upregulated DEPs interact with more than 10 proteins, whose functions are summarized in Table 3. These proteins participate in many important biological processes, including salicylic acid signaling pathway, autophagy, chloroplast development, circadian rhythm as well as many important metabolic pathways, including biosynthesis of fatty acid, ethylene, alkaloid, lignin, demonstrating the important roles played by these proteins.

**Table 3:** Function analysis of the proteins with more than 10 interacting partners in the PPI network

Protein node	Gene name	Enriched GO Term	Degree	Function	Reference
AT1G75270	Dehydroascorbate reductase 2 (DHAR2)	GO:0009407	28	activating the salicylic acid pathway; modulating the redox states of ascorbate–glutathione cycle	Ball et al. (2004); Noshi et al. (2017); Rahantaniaina et al. (2017)
AT4G38630	Multiubiquitin chain binding protein 1 (MBP1)	GO:0006458; GO:0006096; GO:0006090; GO:0046835; GO:0046500; GO:0046086; GO:0019318	23	autophagic receptor; promoting the ubiquitin-mediated degradation of the oxidized proteins degradation	Kurepa et al. (2008)
AT2G28000	Chaperonin 60 Alpha (CPN60A)	GO:0006986; GO:0006458 GO:0051084; GO:0061077	17	chloroplast development; plant response to abiotic and biotic stress	Ke et al. (2017); Suzuki et al. (2009)
AT3G13470	Chaperonin 60 Beta (CPN60B)		14		
AT2G21660	Glycine-rich RNA-binding protein 7 (GR-RBP7)	GO:0006458; GO:0006096; GO:0051084; GO:0050667 GO:0046500; GO:0019318 GO:0061077	14	Stress response; innate immune response; circadian rhythm; flower time	Fu et al. (2007); Kwak et al. (2013); Meyer et al. (2017)
AT1G02500	S-adenosylmethionine synthetase 1 (SAM1)	GO:0006096; GO:0006090; GO:0050667; GO:0046500; GO:0061077	14	Ethylene biosynthesis; lignin deposition, alkaloid biosynthesis	Belbahri et al. (2000); Jin et al. (2017)
AT1G79750	NADP-malic enzyme 4 (NADP-ME4)	GO:0006458; GO:0006096 GO:0006090; GO:0046500; GO:0061077	12	Fatty acid biosynthesis	Wheeler et al. (2005)

## Discussion

As an important component of ethylene signaling pathway transcription factor of ERF1 plays important roles in activating the ethylene signaling pathway through regulating ethylene responsive genes expression in response to environmental stresses. Our research reveals several new mechanisms for regulating plant response to drought stress by ERF1 protein through regulating chloroplast function.

### TERF1 negatively regulates the expression of genes related with photosynthesis

Our results show that overexpression of TERF1 negatively regulates the expression of genes related with photosynthetic light reaction at both transcriptional and translational level, which may result from several important mechanisms. Firstly, our previous research showed that TERF1 significantly repressed the genes encoding the chloroplast transcriptional apparatuses under drought stress condition (Wu et al. 2018). Secondly, a recent study proves that plant response to light needs the degradation of Ethylene-Insensitive3 (EIN3), an upstream activator for ERF1, by phyB (Liu et al. 2017a, b). So overexpression of TERF1 may constitutively repress plant response to light, leading to the repression of genes encoding PhANGs, as it acts downstream of EIN3. Thirdly, TERF1 has also been found to activate the ABA signaling pathway (Zhang et al. 2005) and induce MPK6 (Additional file 11: Figure S3), which also contribute to the repression of the genes related with photosynthetic light reaction (Yamburenko et al. 2015). Finally, the upregulation of carbon fixation and repression of Pi uptake can also lead to the repression of PhANGs (Hausler et al. 2014; Zhang et al. 2014).

Under stress condition the repression of PhANGs in light will lead to the accumulation of reactive oxygen species (ROS), which can induce the expression of stress-related genes (Su et al. 2018). The interaction between ROS production and repression of photosynthesis-related genes orchestrate the trade-off between plant growth and stress tolerance, which can improve plant tolerance of environmental stress (Su et al. 2018).

Photosynthetic genes are encoded by nulear genome and chloroplast genome together. We compared the chloroplast genes expression at both transcriptional level and translational level. Although most of the genes encoded by chloroplast genome are repressed, the correlation between transcript level and protein level is poor. It is reported that translation regulation plays more important roles in chloroplast genes expression than transcription regulation because protein levels vary considerably in response to environmental factors even the corresponding mRNA remain constant (Eberhard et al. 2002). In our study we find that several ribosome genes are both repressed at transcriptional and translational level, which represents a key limit factors for the translational repression for the chloroplast genes (Beligni et al. 2004). Different factors regulate translation of chloroplast genes, so how TERF1 regulates this process deserves future research.

### TERF1 may regulate the interaction between chloroplast and mitochondria

It has been recognized that there exists communication between chloroplast and mitochondria (Raghavendra and Padmasree 2003). From the results we find some important clues for the interaction between chloroplast and mitochondria in TERF1 tobacco that may improve plant tolerance of drought stress. (1) Upregulation of the biosynthesis of valine, leucine and isoleucine not only provide important osmolytes under abiotic stress condition but also can promote oxidization of them in mitochondria, which can provide ATP for photosynthetic carbon fixation under environmental stress conditions (Joshi et al. 2010). As TERF1 significantly repressed ATPase (Additional file 6: Table S4), upregulation of valine, leucine and isoleucine biosynthesis may provide an alternative way to supplement the energy for carbon fixation and cytosol demand under drought stress condition. (2) A key pathway “oxoacid metabolic process” is enriched for the upregulated DEPs, which has been proven to be correlated with a modified TCA cycle in mitochondrial and provides carbon skeleton for N assimilation in chloroplast under environmental stress condition (Padmasree et al. 2002). Then how TERF1 regulates mitochondrial function and the communication between chloroplast and mitochondrial deserves future research.

### TERF1 regulates plant nutrition utilization to improve plant tolerance of drought stress

Our study has shown that TERF1 can regulate plant utilization of nutrition, which can contribute the improvement of plant tolerance of environmental stress. Firstly, the upregulation of the genes related with carbon fixation positively regulates plant response to different environmental stresses and promotes plant growth in the following darkness (Prasch et al. 2015; Fernandez et al. 2017). Secondly, the enhancement of N utilization can improve plant tolerance of environmental stresses and maintain cell membrane stability and leaf tissue integrality (Chang et al. 2016). Finally, the reduction of Pi utilization can repress the expression of PhANGs, which may finally lead to the upregulation of stress-related genes (Su et al. 2018).

### TERF1 can regulate nuclear gene expression in more diversified ways

Previously TERF1 was found to regulate nuclear gene expression through binding the *cis*-element of DRE and GCC box. Our study reveals new potential mechanisms of TERF1 in regulating nuclear gene expression. Firstly, TERF1 can regulate nuclear gene expression through different retrograde signals derived from chloroplast. We have confirmed the interaction between ethylene signaling and retrograde signaling mediated by TERF1 through Q-PCR method, which regulates the expression of PhANGs, plastid redox-associated nuclear genes (PRANGs), singlet oxygen responsive genes (SORGs) (Wu et al. 2018). These retrograde signals not only regulate nuclear gene expression but also connect with different signaling pathways in response to environmental stress as described above. Secondly, TERF1 can regulate gene expression not only at transcriptional level but also at posttranscriptional level. Our analysis shows that TERF1 can regulate Pi uptake at posttranscriptional level through regulating different miRNA. The detailed mechanism of TERF1 regulating nuclear gene expression at posttranscriptional level deserves detailed research in the future, which can enrich our knowledge about ethylene signaling pathway.

## Conclusions

In this study we compared the chloroplast proteome between WT and TERF1 tobacco under drought stress condition when the RWC of WT tobacco was significantly lower than the transgenic tobacco. Our results showed significant difference on the proteins related with photosynthesis, carbon fixation, nutrition utilization between WT and TERF1 tobacco, which provide some new insights into the function of TERF1 and ethylene signaling pathway. This proteome analysis also partially confirmed our previous report about the retrograde signaling mediated by TERF1 (Wu et al. 2018), which is a new mechanism for ERF1 protein regulating nuclear gene expression. We also provide new clues for further research on ethylene signaling and ERF1 protein.

## Materials and methods

### Plant materials and drought stress treatment

*Nicotiana tabacum* L. cv. NC89 was used for *Agrobacterium*-mediated transformation with construct of PROKII containing the ORF of TERF1 driven by 35S promoter (Wu et al. 2018).

Transgenic and WT seeds were first germinated in Petri dish with a moist filter paper at the bottom. After germination seedlings with unanimous growth status were selected and transferred to the soil. The growth condition for the plant is as followings: 28 °C during day time (300 μmol m^−2^ s^−1^) for 16 h, 23 °C at night time for 8 h. All WT and transgenic tobaccos were kept in the same pot to keep unanimous growth condition. Natural dehydration stress was applied for WT and transgenic tobacco at 30 days after germination and relative water content was measured. After 20 days there was significant difference between WT and transgenic tobacco (Additional file 1: Figure S1) and leaves and roots were harvested for further proteome and gene expression analysis. The latest growing leaves were harvested for analysis to minimize the growth difference owing to the drought stress. WT tobaccos under drought stress condition were utilized as control. In this study the proteome and gene expression analysis was applied with three replicates for WT and transgenic tobacco.

### Chloroplast isolation and protein extraction

About 10 g leaves were grinded in 100 mL ice cold grinding medium [50 m MHEPES–KOH (pH 8.0), 330 mM sorbitol, 2 mM EDTA-Na_2_ (pH 8.0), 5 mM ascorbic acid, 5 mM cysteine, 0.05% bovine serum albumin (BSA)]. Filter the homogenate through a double layer of nylon cloth (22 μm). Collect crude chloroplasts by centrifugation for 3 min at 1300*g*. Resuspend the crude chloroplast pellet in wash medium [50 mM HEPES–KOH (pH 8.0), 330 mM sorbitol, 2 mM EDTA-Na_2_ (pH 8.0)] by swirling the suspension. Load the resuspended chloroplast on a Percoll step cushion [PF Percoll (40% or 85%), 0.5 mM EDTA, 50 mM HEPES-KOH (pH 8.0), 330 mM sorbitol] and spin for 10 min at 3750 g in a swing-out rotor. Collect the intact chloroplasts from the 40/85% Percoll interface using a pipet and add wash medium to dilute the Percoll. Spin the chloroplasts for 3 min at 1200 g. Remove the supernatant. The pellets are intact chloroplasts.

### Chloroplast protein extraction

Suspend the chloroplast samples in protein extraction buffer (1.52 g thiourea, 0.3 g 3-[(cholamidopropyl) dimethylammonio]-1-propanesulfonate (CHAPS), 2 mL water, 1 mL 1 M Tris–HCl (pH 8.0), 2 mL 50% glycerol, 0.2 mL Triton X-100, 0.2 mL 1 M DTT, 3.75 mL 8 M urea) and sonicate. Centrifuge the homogenate at 12,000*g* for 5 min at 4 °C and transfer the supernatant to a new tube. Add 1/10 volume of 100% trichloroacetic acid (TCA) to the supernatant and mix. Incubate the solution on ice for 15 min. Centrifuge the homogenate at 10,000*g* for 5 min at 4 °C and discard the supernatant. Add 1 mL ice-cold acetone to the pellet and mix. Centrifuge the homogenate at 10,000*g* for 10 min at 4 °C. Discard the supernatant and resuspend it in 20 μL dissolution buffer and 1 μL denaturant (2% SDS) supplied by iTRAQ kit.

### iTRAQ labeling and strong fractionation by cation exchange chromatography

Add iTRAQ labeling reagents to each protein sample and incubate for 1 h at 37 °C. A 100 μL aliquot of water was added to stop the labeling reaction. A 1 μL aliquot of sample was removed from each group to test labeling and extraction efficiency, and the sample was subjected to a matrix assisted laser desorption ionization procedure after Ziptip desalting. The six sample groups were pooled and vacuum-dried. Each pool of mixed peptides was lyophilized and dissolved in solution A [2% acetonitrile and 20 mM ammonium formate (pH 10.0)]. Then, the samples were loaded onto a reverse-phase column (Luna C18, 4.6 × 150 mm; Phenomenex, Torrance, CA, USA) and eluted using a step linear elution program: 0–10% buffer B [500 mM KCl, 10 mM KH_2_PO_4_ in 25% acetonitrile, (pH 2.7)] for 10 min, 10–20% buffer B for 25 min, 20–45% buffer B for 5 min, and 50–100% buffer B for 5 min at a flow rate of 0.8 mL/min. The samples were collected each min and centrifuged for 5–45 min. The fractions (about 40) collected were finally combined into 10 pools and desalted on C18 Cartridges (Empore™ standard density SPE C18 Cartridges, bed I.D. 7 mm, 3 mL volume; Sigma, St. Louis, MO, USA).

### LC-electrospray ionization-MS/MS analysis

The reconstituted peptides were analyzed with the Q-Exactive mass spectrometer (Thermo Fisher Scientific, Waltham, MA, USA) coupled with a nano high-performance liquid chromatography system (UltiMate 3000 LC Dionex, Thermo Fisher Scientific). The peptides were loaded onto a C18-reversed phase column (3 μm-C18 resin, 75 μm × 15 cm) and separated on an analytical column (5 μm C18 resin, 150 μm × 2 cm; Dr. Maisch GmbH, Ammerbuch, Germany) using mobile phase A: 0.5% formic acid [FA]/H_2_O and B: 0.5% FA/ACN at a flow rate of 300 nL/min, using a 150 min gradient. Spectra were acquired in data-dependent mode. The 10 most intense ions selected for MS scanning (300–1800 m/z, 60,000 resolution at m/z 400, accumulation of 1 × 106 ions for a maximum of 500 ms, 1 microscan). The isolation window was 1.3 m/z, and the MS/MS spectra were accumulated for 150 ms using an Orbitrap. MS/MS spectra were measured at resolution of 15,000 at m/z 400. Dynamic precursor exclusion was allowed for 120 s after each MS/MS spectrum measurement and was set to 17,500 at m/z 200. Normalized collision energy was 30 eV and the underfill ratio, which specifies the minimum percentage of the target value likely to be reached at the maximum fill time, was defined as 0.1%. The instrument was run with peptide recognition mode enabled.

### Sequence database search

The raw mass data were processed for the peptide data analysis using Proteome Discoverer 1.4 (ver.1.4.0.288; Thermo Fisher Scientific) with a false discovery rate [FDR = N(decoy)*2/((N(decoy) + N(target))) < 1% and expected cutoff or ion score < 0.05 (with 95% confidence] for searching the Uniprot Nicotiana tobacco Complete Proteome database. Protein probabilities were assigned using the Protein Prophet algorithm, and proteins with at least two unique peptides were identified. The following options were used to identify the proteins: peptide mass tolerance = ± 15 ppm, fragment mass tolerance = 20 mmu, enzyme = trypsin, max missed cleavage = 2, fixed modification: carbamidomethyl (C), iTRAQ8plex (N-term), iTRAQ8plex (K), iTRAQ8plex (Y), variable modification: Oxidation (M), Acetyl (Protein N-term).

### RNA isolation and reverse transcription

Total RNA and miRNA were extracted from about 200 mg leaf and 500 mg root using RNAiso Plus Kit and RNAiso for small RNA kit (Takara Biomedical Technology, Japan), respectively. RNA integrity was confirmed by electrophoresis and RNA quantity was determined by NanoDrop *ND*-*1000* spectrophotometer (NanoDrop Technologies, Wilmington, DE, USA). Total RNA and miRNA were utilized for reverse transcription using Prime Script™ RT reagent Kit with gDNA Eraser and Mir-X™ miRNA First Strand Synthesis Kit (Takara Biomedical Technology, Japan), respectively.

### Quantitative real-time polymerase chain reaction (qRT-PCR)

qRT-PCR was performed with 3 replicates by *CFX96* real-time PCR detection system (Bio-Rad, Hercules, USA). SYBR^®^ Premix Ex Taq™ II (Tli RNaseH Plus) kit and Mir-X™ miRNA qRT-PCR SYBR^®^ Kit (Takara Biomedical Technology, Japan) was used for mRNA and miRNA qRT-PCR, respectively. Tobacco *L25* (GenBank accession number L18908) and *ERF1α* (GenBank accession number AF120093) were utilized to normalize the mRNA genes expression value. 5S RNA was utilized to normalize the miRNA genes expression value. *Beacon designer 8.0* was utilized to design the gene specific primers (Additional file 12: Table S1). The qPCR reaction was performed as following: 95 °C for 5 min, followed by 40 cycles (95 °C for 30 s, 60 °C for 30 s, and 72 °C for 30 s), followed by melting curve analysis: at 50 °C for 30 s and then at 65–95 °C (0.5 °C increments, 5 s for each). The chloroplast genome sequences of tobacco were retrieved from NCBI database (Accession number: NC_001879.2). The nuclear-encoded genes sequences of tobacco were retrieved from *Sol Genomics Network* database according to the latest genome annotation (https://solgenomics.net/).

### Relative water content analysis

Relative water content (RWC) was calculated by the formula: RWC (%) = (FW − DW)/(TW − DW) × 100. FW: fresh weight, TW: turgid weight, DW: dry weight.

## Additional files

**Additional file 1: Figure S1.** Comparison of relative water content (RWC) under drought stress condition.

**Additional file 2: Table S9.** Chloroplast proteins identified in WT and TERF1 tobacco by iTRAQ method.

**Additional file 3: Table S2.** Identification and quantification of chloroplast proteins in WT and TERF1 tobacco.

**Additional file 4: Figure S2.** Principal component analysis of the chloroplast proteome in WT and TERF1 tobacco.

**Additional file 5: Table S3.** Proteins up regulated by TERF1 under drought stress condition.

**Additional file 6: Table S4.** Proteins downregulated by TERF1 under drought stress condition.

**Additional file 7: Table S5.** Gene ontology enrichment analysis for the upregulated proteins by TERF1.

**Additional file 8: Table S6.** Gene ontology analysis for the downregulated proteins by TERF1.

**Additional file 9: Table S7.** Identification and quantification of the chloroplast genome encoded proteins in WT and TERF1 tobacco.

**Additional file 10: Table S8.** Expression analysis of the chloroplast genome encoded genes.

**Additional file 11: Figure S3.** Expression analysis of MPK6 between WT and TERF1 tobacco. Means ± SDs, n = 3; * - significantly different at 5% level of probability.

**Additional file 12: Table S1.** Primers Used in This Research.

## Abbreviations

ABA: abscisic acid
DEPs: differentially expressed proteins
ERF: ethylene response factor
GO: gene ontology
GS: glutamine synthetase
GSLA: gene set linkage analysis
N: nitrogen
NR: nitrate reductase
Pi: phosphorus
PPI: protein–protein interaction
RWC: relative water content
TERF1: tomato ethylene responsive factor 1
Trx: thioredoxin
WT: wild type
