# Supplementary figures and images for: Chloroplast proteome analysis of Nicotiana tabacum overexpressing TERF1 under drought stress condition
Source: Bot Stud. 2018 Oct 29;59:26. doi: 10.1186/s40529-018-0239-5 (PMC6206318; doi:10.1186/s40529-018-0239-5)

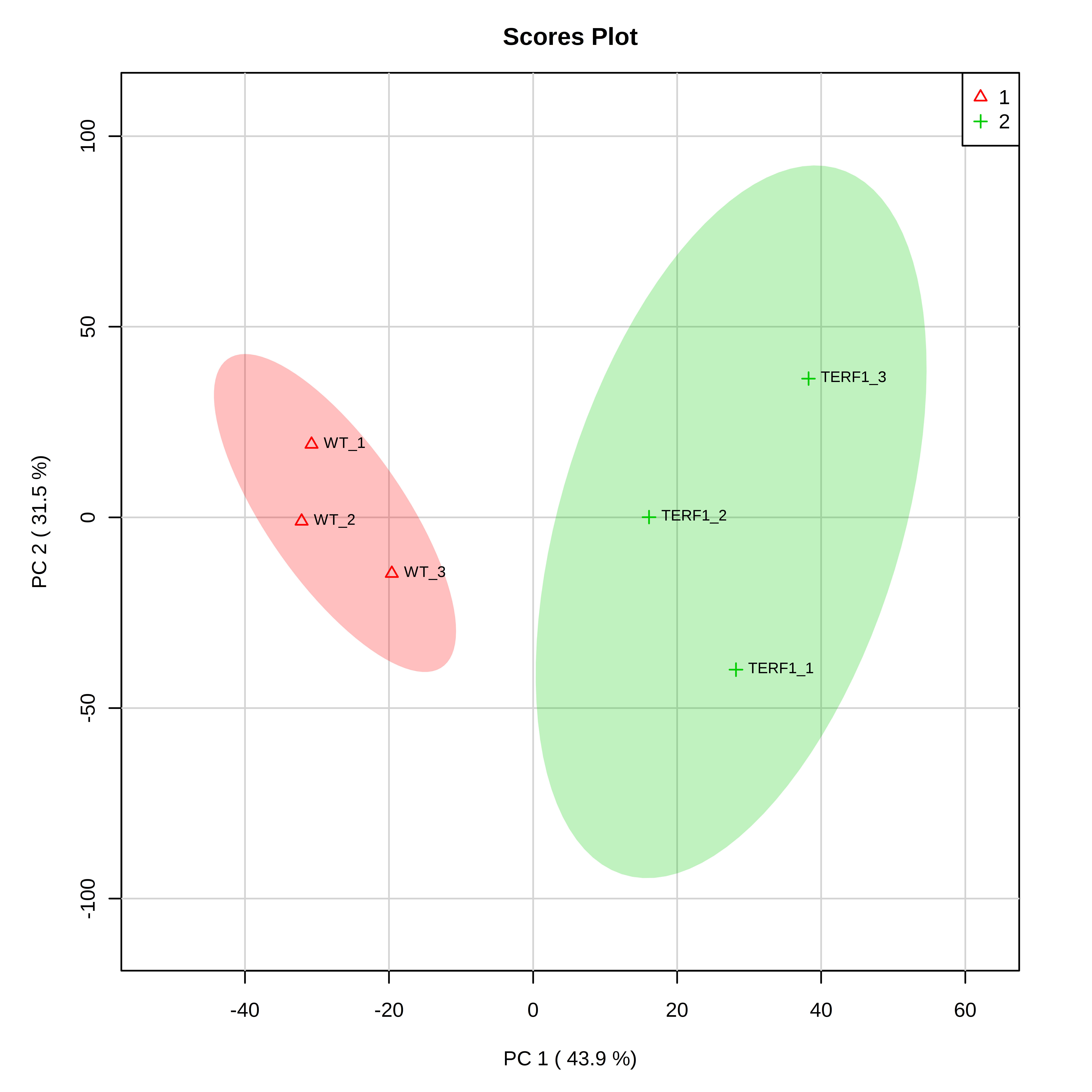

Supplement: Supplementary file 4 — Additional file 4: Figure S2. Principal component analysis of the chloroplast proteome in WT and TERF1 tobacco. [file 40529_2018_239_MOESM4_ESM.tif]
